# Examination of metacognitions and functionality in agoraphobia without comorbidities

**DOI:** 10.1186/s12888-025-07003-y

**Published:** 2025-05-26

**Authors:** Ulaş Korkmaz, Fatma Gül Helvacı Çelik, Meltem Hazel Şimşek

**Affiliations:** Department of Psychiatry, Giresun University, Giresun, Turkey

**Keywords:** Agoraphobia, Functionality, Metacognition

## Abstract

**Background:**

Although agoraphobia is considered an independent diagnosis, the literature is limited in studies examining it alone. This study aims to investigate the relationship between agoraphobia, metacognitive beliefs, and functionality and to increase interest in studies on agoraphobia by contributing to areas that are missing in the literature.

**Methods:**

Seventy healthy controls without any psychiatric disorder and seventy individuals with agoraphobia without comorbidities were included in the study. Data regarding sociodemographic characteristics, agoraphobic symptoms, metacognitive beliefs, functionality, depression, and anxiety levels were collected from the participants. Comparisons were made between the groups. Mediation analysis was performed by adjusting for sociodemographic and clinical variables to determine the mediating role of metacognitions in the effect of agoraphobia severity on functionality.

**Results:**

The agoraphobia group scored significantly higher than controls on pathological metacognitive beliefs, anxiety, and depression measures (*p* < 0.05), and showed more severe impairment in all functional domains (*p* < 0.05). Metacognitive belief levels were positively correlated (*p* < 0.001) with agoraphobia severity (*r* = 0.570) and functional impairment (*r* = 0.537). Mediation analysis indicated that metacognitive beliefs significantly mediated the impact of agoraphobia severity on functionality, accounting for approximately 26% of the total effect.

**Conclusions:**

Metacognitive beliefs mediated the relationship between the severity of agoraphobia and functional impairment (mediation effect accounting for 26%). Metacognitive processes play a key role in how agoraphobia severity translates into functional impairment. Targeting dysfunctional metacognitive beliefs in treatment may improve functional outcomes in agoraphobia. These findings highlight the need for further longitudinal and experimental studies focusing on agoraphobia without comorbid conditions.

## Introduction

Anxiety disorders are an important public health problem due to their worldwide prevalence and their adverse effects on individuals' quality of life [1]. Among these disorders, agoraphobia is characterized by a fear of being in places where it is difficult to stay or where help is out of reach and an intense anxiety response to these situations [2]. Agoraphobia was first described as a neurosis by the German psychiatrist Carl Westphal [3] in 1871. Westphal used this term to describe individuals who experience intense anxiety and avoidance behavior in crowded, open spaces or social situations. Today, the definition of agoraphobia is broader. Such anxiety can narrow the individual's living space, lead to dependency on others, and pave the way for the development of accompanying mental disorders such as depression [4].

Agoraphobia was considered within the scope of panic disorder in the Diagnostic and Statistical Manual of Mental Disorders (DSM)-IV and before. However, with DSM-5, it was considered an independent diagnosis and took its place in the anxiety disorders class [5]. This regulation was made possible by the increasing number of studies supporting the evaluation of agoraphobia as an independent psychiatric disorder [4]. Agoraphobia is relatively new as a separate diagnosis compared to other anxiety disorders. In parallel with this, it is noteworthy that studies examining agoraphobia alone are limited in the literature. There are mostly studies on panic disorder classification (with and without agoraphobia). However, agoraphobia is now a separate disorder and is not always found in comorbid conditions [6]. Genetic predisposition, irregularities in brain neurotransmitters, and environmental factors play important roles in the etiology of agoraphobia. In addition to biological mechanisms such as imbalances in the serotonergic system and increases in cortisol levels, cognitive and metacognitive processes are also determinants of the development and maintenance of this disorder [7].

Functionality refers to the individual's capacity to sustain daily physical, psychological, and social life. In psychiatry, functionality is a fundamental concept for evaluating the effects of disorders on the individual and determining treatment goals [8]. As in other anxiety disorders, a significant impairment in the functionality of individuals can be observed in agoraphobia [9]. In severe cases, individuals may become unable to leave their homes, which can have costs at both individual and societal levels. Individuals diagnosed with agoraphobia have a high rate of other comorbid psychiatric disorders, and approximately 15% of these individuals have suicidal thoughts or behaviors [2]. However, there is no clear information in the literature regarding functionality in agoraphobia without comorbidities.

As in all anxiety disorders, cognitive distortions and inferences have an important place in agoraphobia. While the cognitive behavioral model focuses on irrational thoughts about situations that individuals perceive as dangerous, the metacognitive model draws attention to the effect of individuals' beliefs about their thoughts that maintain anxiety [10]. The metacognitive model proposes that individuals' beliefs about their thoughts trigger maladaptive regulation strategies. Although these strategies may seem to reduce anxiety, they cause distress to persist and intensify [11]. In this regard, the metacognitive model expands our understanding of the etiology, maintenance, and treatment of any type of anxiety disorders [12]. In particular, excessive focus on negative thoughts and beliefs may contribute to the maintenance of anxiety disorders. In agoraphobia, individuals often develop metacognitive beliefs that support their fear, such as moving away from their safe place or losing control [10]. This may increase attentional bias and avoidance behaviors towards the threat. [13]. Such dysfunctional metacognitions likely contribute not only to heightened anxiety but also to functional impairment by narrowing individuals’ life activities due to distorted biases. In fact, higher levels of pathological metacognitive beliefs have been associated with poorer social functioning in clinical samples [14].

Even after DSM-5, research on agoraphobia without comorbidities is quite scarce. Agoraphobia is still being studied as a feature of panic disorder. In addition, the lack of original research on the relationship between agoraphobia and metacognition in the literature is striking. In particular, studies evaluating the effects of metacognitive processes on the DSM-5 agoraphobia definition appear to be limited. Likewise, studies specifically addressing the effect of agoraphobia without comorbidities on functionality are also limited. According to the literature, agoraphobia is associated with functional impairment. However, there is no information about the role of metacognition, another factor that causes functional impairment, in the relationship between agoraphobia and functionality. In this context, investigating the relationship between metacognitive beliefs and symptom severity and functionality in individuals diagnosed with agoraphobia may provide new insights into the etiology and treatment strategies of the disorder. In addition, such studies can potentially increase interest in studies on agoraphobia by contributing to areas that remain missing in the literature.

Our hypotheses are as follows:

- H1) Functionality is more impaired in individuals with agoraphobia than in healthy controls.
- H2) Pathological metacognitive beliefs are more common in individuals with agoraphobia than in healthy controls.
- H3) Metacognitive processes mediate the relationship between agoraphobia severity and functionality.

## Methods

### Participants and procedure

The patient group consisted of individuals who applied to the psychiatry outpatient clinic of Giresun University Training and Research Hospital, affiliated with the Ministry of Health of the Republic of Turkey, and were diagnosed with agoraphobia according to DSM 5. The inclusion criteria for the patient group were 1) Having a diagnosis of agoraphobia, 2) Being between 18–65 years old, 3) Having ability to read and understand the study questionnaires, and 4) Being willing to participate in the study. The exclusion criterion for the patient group was having a medical disease that could affect the mental state of the patients or a psychiatric disorder other than agoraphobia. The healthy control (HC) group consisted of healthy individuals with sociodemographic characteristics similar to the patient group. The inclusion criteria for the HC group were 1) Being between 18–65 years old, 2) Having ability to read and understand the study questionnaires, and 3) Being willing to participate in the study. Exclusion criteria for the HC group included the presence of any medical condition that could affect the individual’s mental state or any current psychiatric disorder. All participants underwent a clinical evaluation by a psychiatrist. The Structured Clinical Interview for DSM-5 was administered to confirm the agoraphobia diagnosis (and the absence of other psychiatric diagnoses) in patients, and to verify that control participants had no psychiatric diagnosis. Participants were also screened for medical conditions via history and examination; any neurological or chronic medical illnesses known to impact mental health (for example, epilepsy, organic mental disorders, or thyroid dysfunction) led to exclusion from the study.

Both groups comprised 70 participants each. After obtaining consent, a sociodemographic data form and a functioning assessment short test (FAST) were filled out for the participants. Then, the agoraphobia scale (AS), metacognition questionnaire-30 (MCQ-30), Beck anxiety inventory (BAI), and Beck depression inventory (BDI) were applied to the participants. The research data was collected between July 2023 and January 2025.

The Giresun Training and Research Hospital Clinical Research Ethics Committee approved the research, decision number 17.07.2023/03. The research was conducted in accordance with the Declaration of Helsinki.

### Measures

#### Sociodemographic data form

The researchers created this form to determine the participants' sociodemographic characteristics. The form contains information about the participants' ages, years of education, gender, marital status, employment status, place of residence, lifestyle, socioeconomic status, family history of psychiatric disorders, and previous suicide attempts.

Agoraphobia scale (AS).

The AS is based on DSM-5 and is used to measure the severity of agoraphobia. It consists of ten items ranging from 0 to 4 points. It is a self-report scale that reflects the severity of agoraphobia symptoms over the past week. The scale has no cut-off point. The higher the scores obtained from the scale, the higher the severity of agoraphobia. The scale can be scored at a minimum of 0 points and a maximum of 40 points. The Cronbach alpha internal consistency coefficient of the scale was found to be 0.95 [15].

#### Metacognition questionnaire-30 (MCQ-30)

MCQ-30 was developed by Wells and Cartwright-Hatton [16], and its final form, consisting of 30 items, was created. MCQ-30 is a 4-point Likert-type self-report scale ranging from 1 to 4 points. The scale does not have any cut-off score. Higher total scores indicate more pathological metacognitive beliefs; total scores range from 30 to 120. In the validity and reliability study conducted in Turkey, the internal consistency coefficient was found to be 0.86 [17].

#### Functioning assessment short test (FAST)

FAST, developed by Rosa et al. [18] to assess functionality, is a scale the interviewer applies to participants. It consists of 24 questions on a four-point Likert scale ranging from 0 to 3. It assesses six dimensions: autonomy, occupational functioning, cognitive functioning, financial issues, interpersonal relationships, and leisure time. Additionally, the total score can be used to assess functionality. A total score (ranging from 0 to 72) can be calculated, with higher scores indicating greater functional impairment. In the Turkish validity and reliability study, Cronbach's α value was determined as 0.96 [19].

#### Beck anxiety inventory (BAI)

The BAI was developed by Beck et al. [20]. It is a self-report scale comprising 21 questions on a 4-point Likert scale ranging from 0 to 3 points. Total scores range from 0 to 63, with higher scores reflecting more severe anxiety. Scores are interpreted as follows: 0–7 = minimal anxiety, 8–15 = mild anxiety, 16–25 = moderate anxiety, and 26–63 = severe anxiety. In the Turkish validity and reliability study, Cronbach's α value was determined as 0.93 [21].

#### Beck depression inventory (BDI)

The BDI is a self-report scale with 21 questions on a 4-point Likert-type scale ranging from 0 to 3. Total scores range from 0 to 63, and higher scores reflect more severe depression. Scores are interpreted as follows: 0–9 = minimal depression, 10–16 = mild depression, 17–29 = moderate depression, and 30–63 = severe depression. Beck et al. [22] developed the BAI. In the validity and reliability study conducted in Turkey, the internal consistency coefficient was found to be 0.8 [23].

### Statistical analyses

Statistical analyses were performed using IBM SPSS Statistics 27. Whether the data had a normal distribution based on skewness and kurtosis values was decided. If the skewness and kurtosis values ​​were between -1.5 and + 1.5, the data were considered to have a normal distribution [24]. The study included two independent groups to compare. Therefore, the Chi-square test was used to compare categorical variables, the Student's t-test was used to compare numerical variables with normal distribution, and the Mann–Whitney U test was used to compare numerical variables without normal distribution. The correlations between the variables were evaluated using Pearson or Spearman correlation analyses, depending on whether the data were normally distributed. Mediation analysis was performed to examine the effect of metacognitive beliefs on the relationship between agoraphobia symptom severity and level of functional impairment. In the model established for this purpose, it was assumed that the severity of agoraphobia affects metacognitive beliefs, and metacognitive beliefs affect functional impairment. Mediation analyses were performed with IBM SPSS Statistics Process macro. The regression method based on the bootstrap method, and the 5000 resampling option were preferred. Adjustments were made for sociodemographic and clinical variables in the mediation analysis. The statistical significance level (p) was accepted as 0.05 for all tests.

## Results

The mean duration of disorder in the agoraphobia group was 5.43 (SD: 7.2) years. The mean agoraphobia severity score was 21.81 (SD: 10.73) points. Seventeen patients (24.3%) had their first psychiatric visit. Six patients (8.6%) had a history of inpatient treatment. Forty-four patients (62.9%) were not receiving any treatment at the time of evaluation.

No statistically significant difference was found between the agoraphobia and HC groups in terms of age, gender, marital status, place of residence, and lifestyle. The HC group had a higher education level, proportion of employed people, and socioeconomic status. History of suicide attempts in the past and history of psychiatric disorders in their families were more common in the agoraphobia group than in the HC group. The comparison of the groups' sociodemographic characteristics is shown in Table 1.

**Table 1:** Comparison of sociodemographic characteristics

	**Agoraphobia (*n* = 70)**	**HC** **(*n* = 70)**	**Statistics**	***p***
	Median (Q1-Q3) / Mean ± SD / n (%)			
**Age (years)**	37.83 ± 11.82	37.39 ± 10.41	t = 0.235	0.814
**Duration of education (years)**	12 (10.50–16)	16 (13.75–16)	U = 1640.500	** < 0.001**
**Gender**			X^2^ = 0.463	0.496
Female	29 (41.4%)	37 (52.9%)		
Male	41 (58.6%)	33 (47.1%)		
**Marital status**			X^2^ = 0.122	0.726
Single	25 (35.7%)	27 (38.6%)		
Married	45 (64.3%)	43 (61.4%)		
**Employment status**			X^2^ = 34.192	** < 0.001**
Employed	29 (41.4%)	62 (88.6%)		
Unemployed	41 (58.6%)	8 (11.4%)		
**Place of residence**			X^2^ = 1.087	0.297
Village-town	17 (24.3%)	12 (17.1%)		
City center	53 (75.7%)	58 (82.9%)		
**Lifestyle**			X^2^ = 3.451	0.063
Living alone	7 (10%)	15 (21.4%)		
Living with someone	63 (90%)	55 (78.6%)		
**Socioeconomic status**			X^2^ = 9.882	**0.007**
Low	11 (15.7%)	1 (1.4%)		
Middle	51 (72.9%)	63 (90%)		
High	8 (11.4%)	6 (8.6%)		
**Family history of psychiatric disorders**			X^2^ = 5.829	**0.016**
No	43 (61.4%)	56 (80%)		
Yes	27 (38.6%)	14 (20%)		
**Previous suicide attempts**			X^2^ = 9.618	**0.003**
No	61 (87.1%)	100 (100%)		
Yes	9 (12.9%)	0 (0%)		

*n* Number of samples, *p*: Significance level, *Q1* First quartile, *Q3* Third quartile, *SD* Standard deviation

The comparison of the agoraphobia group and the HC group in terms of scales related to metacognitive beliefs, functional impairment, anxiety, and depression levels is given in Table 2. Higher pathological metacognitive beliefs, anxiety, and depression scores were found in the agoraphobia group compared to the HC group. Additionally, more significant impairment was observed in all areas of functionality in the agoraphobia group.

**Table 2:** Comparison of clinical variables

	**Agoraphobia (*n* = 70)**	**Control** **(*n* = 70)**	**Statistics**	***p***
	Median (Q1-Q3) / Mean ± SD			
**MCQ-30**	70.39 ± 15.93	64.66 ± 13.5	t = 2.295	**0.023**
**FAST**	26.76 ± 17.96	13.99 ± 11.55	t = 5.004	** < 0.001**
Autonomy	4.43 ± 3.5	2.13 ± 2.13	t = 4.699	** < 0.001**
Occupational functioning	5.5 (1–10)	1 (0–4)	U = 1396.500	** < 0.001**
Cognitive functioning	5.46 ± 5.15	3.99 ± 3.21	t = 2.027	**0.045**
Financial issues	1 (0–4)	0 (0–2)	U = 1970.500	**0.029**
Interpersonal relationships	6.61 ± 5.15	3.4 ± 3.82	t = 4.197	** < 0.001**
Leisure time	2.71 ± 2.07	1.77 ± 1.84	t = 2.855	**0.005**
**BAI**	29.97 ± 16.01	12.09 ± 10.16	t = 7.891	** < 0.001**
**BDI**	20 (9.75–32.25)	6.5 (2–11)	U = 984.500	** < 0.001**

*n* Number of samples, *p*: Significance level, *Q1* First quartile, *Q3* Third quartile, *SD* Standard deviation,*BAI* Beck anxiety inventory, *BDI* Beck depression inventory, *FAST* Functioning assessment short test, *MCQ-30* Metacognition questionnaire-30

Table 3 reports the results of correlation analyses performed in the agoraphobia group. The correlation analyses found moderate positive correlations between clinical variables.

**Table 3:** Correlation coefficients of clinical variables in the agoraphobia group

	**BDI**	**BAI**	**FAST**	**MCQ-30**
AS	0.431*	0.613*	0.610*	0.570*
MCQ-30	0.473*	0.417*	0.537*	
FAST	0.585*	0.643*		
BAI	0.729*			

^*^p < 0.001

*AS* Agoraphobia scale, *BAI* Beck anxiety inventory, *BDI* Beck depression inventory, *FAST* Functioning assessment short test, *MCQ-30* Metacognition questionnaire-30

**Fig. 1:**
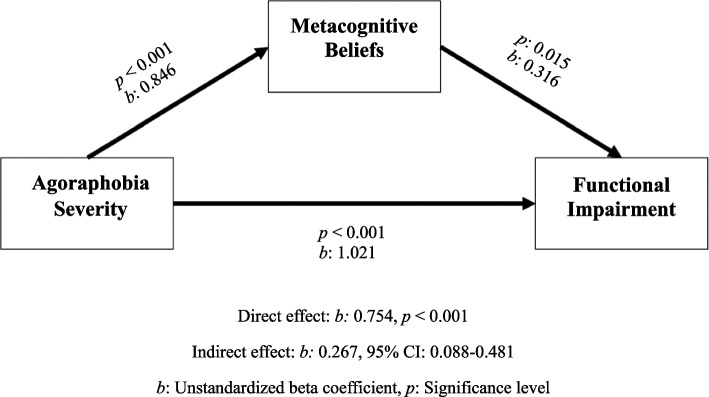
Mediation model of the relationship between agoraphobia and functionality

The model created to show the mediating effect of metacognitive beliefs on the relationship between agoraphobia severity and functionality is visualized in Fig. 1. The model was statistically significant (*p* < 0.001) and explained approximately 43% of the effect on functionality (R^2^ = 0.425). The total effect (*b* = 1.021, 95% CI = 0.700–1.342) and direct effect (*b* = 0.754, 95% CI = 0.377–1.131) of agoraphobia severity level on functional impairment were statistically significant. According to the model, a one-unit increase in agoraphobia severity directly causes a 0.754-unit increase in the level of functional impairment. In addition, the indirect effect of agoraphobia severity on the level of functional impairment through metacognitive beliefs was found to be statistically significant (*b* = 0.267, 95% CI = 0.088–0.481). After adjustment for age, gender, education level, disease duration, anxiety, and depression levels, the total effect of agoraphobia severity level on functional impairment (*b* = 0.558, 95% CI = 0.176–0.941) remained statistically significant, while its direct effect (*b* = 0.350, 95% CI = -0.081–0.780) did not. The mediating effect through metacognitive beliefs remained significant (*b* = 0.209, 95% CI = 0.035–0.428). As a result, it was determined that metacognitions had a mediating role in the effect of agoraphobia severity on functionality (mediation effect accounting for 26%). The full standardized effect size (K^2^) of the mediation effect of metacognitive beliefs is 0.16, indicating a medium-level effect.

## Discussion

This study examined the relationships between agoraphobia, metacognitive beliefs, and functional impairment in the absence of comorbid conditions. Consistent with our hypotheses, individuals with agoraphobia had markedly greater functional impairment (H1) and more pathological metacognitive beliefs (H2) than healthy controls. Furthermore, metacognitive beliefs were shown to mediate the relationship between agoraphobia severity and functional impairment (H3). To our knowledge, this is one of the first studies to demonstrate this mediating role of metacognition in agoraphobia. These findings suggest that even without comorbid psychiatric disorders, agoraphobia is associated with significant dysfunction, and that metacognitive processes play a critical role in this outcome.

Studies on agoraphobia have received relatively limited attention in the literature so far. Probably one of the reasons for this is that agoraphobia is often seen together with other psychiatric diagnoses [25]. Cases of agoraphobia without comorbidities are not common [6]. One of the indicators of this may be that we were able to include seventy patients in our study in approximately 18 months in a busy outpatient psychiatric clinic. Nevertheless, although individuals diagnosed with agoraphobia may exhibit panic-like symptoms, it may exist independently of panic disorder or other psychiatric disorders [6].

In our study, the level of education, the proportion of employed people, and the socioeconomic status were higher in the HC group than in the agoraphobia group. This difference is consistent with agoraphobia worsening functionality [6]. Decreased functionality may be a natural result of a phobia against using vehicles for transportation, being in open, closed, or crowded places, and being alone [5]. We also found a history of past suicide attempts and a family history of psychiatric disorders to be higher in the agoraphobia group. A study reported that agoraphobia was not associated with suicidal thoughts, but the study was conducted with individuals with panic disorder and used a small sample [26]. Another more recent study reported similar rates of suicidal ideation in panic disorder with and without agoraphobia, at 25%. This finding suggests that agoraphobia may be a risk factor for suicide [27]. Agoraphobia may also be associated with suicidal ideation through depression [28]. Childhood neglect is a risk factor for anxiety disorders [29]. Psychiatric disorders of caregivers may increase this neglect [30]. Therefore, our findings regarding suicide attempts and family history appear to be consistent with the literature. However, studies on the causal relationship between agoraphobia and childhood trauma are lacking. Moreover, no study has been found examining the relationship between agoraphobia without comorbidities and suicide.

We found higher levels of pathological metacognitive beliefs, anxiety, and depression in the agoraphobia group compared to the HC group. It was an expected finding that anxiety and depression levels were higher in individuals diagnosed with agoraphobia and that the severity of agoraphobia was correlated with anxiety and depression levels. Agoraphobia goes with emotional-physiological, cognitive, and social problems. The distress caused by the symptoms of agoraphobia and anxiety and depression emerge as phenomena that affect and increase each other [31]. From a cognitive and metacognitive perspective, worries learned from various experiences are maintained by positive or negative attitudes toward these worries, that is, by metacognitive processes. This process, which is initially a coping mechanism against threats, itself creates a perception of threat, leading to a vicious cycle of anxiety. This situation indicates that metacognitive processes have turned into a pathological style [32]. Again, agoraphobic symptoms may occur as a coping mechanism secondary to frightening panic attacks in panic disorder [33]. No specific model has been stated for agoraphobia, but the cognitive-metacognitive model as a transdiagnostic model may also be applicable to agoraphobia. Agoraphobia has long been conceptualized as a fear of fear [34]. This draws attention to the metacognitive nature of agoraphobia. Therefore, the behaviors of worrying about worries and avoiding situations in which worries might arise may explain the essential nature of agoraphobia. Consistent with this model, we found more metacognitive beliefs in the agoraphobia group compared to healthy controls. Moreover, the positive correlation of metacognitions with agoraphobia symptom severity provides clues about the etiology and maintenance of agoraphobia. Metacognitive beliefs are significantly associated with functioning, depression, and anxiety [14]. Therefore, our findings are consistent with previous literature, and we confirmed that this relationship is also valid in individuals diagnosed with agoraphobia. Cognitive behavioral psychotherapies are highly effective in panic disorder with agoraphobia [35]. Metacognitive psychotherapies have also been reported to be effective [36]. However, the mechanisms for the treatment of agoraphobia in this context are unclear. Because the studies were mostly conducted in comorbid conditions. Future experimental and descriptive studies may provide important results regarding the metacognitive model and treatment of agoraphobia.

According to our results, there is more impairment in all areas of functionality in the agoraphobia group. Wittchen et al. [6] stated that agoraphobia without panic attacks affects functioning at least as much as panic disorder, and agoraphobic symptoms worsen functioning more in comorbid conditions. Symptoms related to agoraphobia may prevent patients from living an autonomous life, being in professionally necessary places, and using their cognitive abilities due to intense stress. Additionally, an important feature of agoraphobia is its relationship with impairment in social functioning [37]. Individuals with agoraphobia may experience problems in their interpersonal relationships due to the stress and anxiety they experience, and they may not be able to spare time for enjoyable leisure activities due to their symptoms. In our sample, as the severity of agoraphobia increased, functional impairment also increased. Our findings provide important information that agoraphobia causes a significant decrease in functionality.

Psychiatric disorders, by definition, cause impairment in functioning [5]. However, understanding the causes of functional impairment is important for treatment. Because of the complex nature of psychiatric disorders, relationships always require multifactorial evaluation. In our study, we found that metacognitions mediate the effect of agoraphobia severity on the level of functional impairment. When metacognitions were taken into account, the direct effect of agoraphobia severity was still significant. However, this direct effect did not remain significant after statistical analyses adjusted for sociodemographic and clinical variables. This result indicates that the effect on functionality is determined by factors other than symptom severity. After statistical adjustments, the mediation effect through pathological metacognitions remained significant. This result shows that metacognitions have an important relationship with functional impairment in individuals with agoraphobia and that it is a phenomenon that should be taken into consideration in treatment, especially in terms of increasing functionality in patients. Metacognitive therapies, which are a transdiagnostic method, may also be effective in agoraphobia.

Although our study provides new contributions to the literature as it evaluates agoraphobia without comorbidities, it has some limitations. Our study has a cross-sectional design. Therefore, a cause-effect relationship cannot be established. Our sample is relatively small and was conducted at a single center. This makes it difficult to generalize the results to all patients with agoraphobia. Some sociodemographic differences may have affected the results of the comparison of clinical variables between the two groups. We did not evaluate personality characteristics. Additionally, the scales, which are self-report instruments, may have affected the objectivity of the data.

## Conclusions

We found that functionality was impaired in individuals with agoraphobia without any comorbidity and that these individuals had a pathological metacognitive style. We found that metacognitions were significantly associated with functioning in individuals with agoraphobia. Considering the role of cognitive and metacognitive processes in the etiology and maintenance of agoraphobia, it can be assumed that metacognitive psychotherapies may be a potentially effective method in the treatment of agoraphobia. However, due to the limited number of studies addressing agoraphobia without comorbidities in the current literature, further research is needed. In particular, longitudinal studies examining the effects of metacognitive psychotherapy and metacognitive processes on agoraphobia in more detail may provide more precise results. In addition, comparing the agoraphobia group with other psychiatric disorders in terms of cognitive parameters may contribute to the formation of new perspectives in psychiatric nosology. While our findings emphasize the importance of considering metacognitive processes in treatment approaches, they reveal the lack of isolated studies on agoraphobia and the need for further research.

## Data Availability

Data available on request from the authors.
